# Boolean Modeling of Cellular and Molecular Pathways Involved in Influenza Infection

**DOI:** 10.1155/2016/7686081

**Published:** 2016-02-14

**Authors:** Christopher S. Anderson, Marta L. DeDiego, David J. Topham, Juilee Thakar

**Affiliations:** ^1^Department of Microbiology and Immunology, University of Rochester Medical Center, Rochester, NY 14642, USA; ^2^Department of Biostatistics and Computational Biology, University of Rochester Medical Center, Rochester, NY 14642, USA

## Abstract

Systems virology integrates host-directed approaches with molecular profiling to understand viral pathogenesis. Self-contained statistical approaches that combine expression profiles of genes with the available databases defining the genes involved in the pathways (gene-sets) have allowed characterization of predictive gene-signatures associated with outcome of the influenza virus (IV) infection. However, such enrichment techniques do not take into account interactions among pathways that are responsible for the IV infection pathogenesis. We investigate dendritic cell response to seasonal H1N1 influenza A/New Caledonia/20/1999 (NC) infection and infer the Boolean logic rules underlying the interaction network of ligand induced signaling pathways and transcription factors. The model reveals several novel regulatory modes and provides insights into mechanism of cross talk between NF*κ*B and IRF mediated signaling. Additionally, the logic rule underlying the regulation of IL2 pathway that was predicted by the Boolean model was experimentally validated. Thus, the model developed in this paper integrates pathway analysis tools with the dynamic modeling approaches to reveal the regulation between signaling pathways and transcription factors using genome-wide transcriptional profiles measured upon influenza infection.

## 1. Introduction

Systems virology facilitates deeper understanding of how viruses cause diseases by integrating host-directed approaches to study viral pathogenesis [1]. Genome-wide transcriptional profiling studies have been instrumental in measuring large-scale changes in the host upon viral infections. Pathogenic viruses, in particular influenza virus (IV), frequently cause mild respiratory disease, whereas some strains such as 1918 pandemic virus can cause severe respiratory illness [2, 3]. Understanding underlying molecular signature of IV infection outcome is critical towards designing efficient therapeutics and vaccines. The genome-wide transcriptional profiles of IV pathogenesis have identified several transcription factors (TFs) such as IRF7, STAT1, and NF*κ*B1, and signaling pathways such as RIG-I, MDA5, and Type I IFNs involved in early innate immune responses against IV infections [4, 5]. Transcriptomic studies have also been instrumental in identifying markers associated with severe IV infections, which are generally characterized by an early, sustained, and excessive inflammatory response that is regulated by NF*κ*B, HMGA1, and NFATC4 TFs [6–10]. The identification of pathways and TFs from transcriptomic data is currently achieved by using statistical approaches that combine the expression profiles of genes with the available databases defining the genes involved in the pathways (gene-sets). However, such enrichment techniques do not take into account interactions among pathways responsible for the generation of dynamic response to IV infection.

Combining these high-throughput data with the computational techniques, particularly the ones embedded in the theory of dynamical systems, improves our understanding about the emergent properties of the system that are clinically relevant [11]. Dynamic models can bridge this gap by integrating the static network with a mathematical framework to describe the status of the system over time. Qualitative approaches such as discrete dynamic modeling can be developed for large systems even when knowledge of kinetic parameters is limited [12]. Hence, they are ideal for understanding system-wide high-throughput assays. Particularly, Boolean networks [13] have been used for modeling cellular and intracellular interactions relevant to immunology [14–18]. Moreover, various observations, such as the sigmoidal (S-like) shape of the input-output curves of regulatory relationships and the robustness of biological networks when faced with fluctuations in concentrations and reaction rates, lend support to the applicability of Boolean and other qualitative models [12, 19]. Hence, to study emergent properties of the network underlying antiviral responses induced upon IV infections, we used a combination of laboratory experiments and network-based discrete dynamic modeling approach.

Particularly, we investigate dendritic cell (DC) response to seasonal H1N1 influenza A/New Caledonia/20/1999 (NC) infection and infer the Boolean logic rules underlying the ligand induced signaling pathways and TFs interactions. The model revealed several novel regulatory modes and provides insights into mechanism of cross talk between NF*κ*B and IRF mediated signaling. Additionally, the logic rule underlying the regulation of IL2 pathway that was predicted by the Boolean model was experimentally validated, providing novel insight into the regulation of IL2 pathway.

## 2. Methods

### 2.1. Brief Description of the Dataset and Assembly of the Networks

Raw expression data of seasonal NC infected and mock-infected DCs was obtained from our previous work (GEO ID GSE41067) [5]. Briefly, NC stocks were added into pelleted monocyte-derived DCs at multiplicity of infection (MOI) of 1. RNA was collected at seven time points: before infection and 2, 4, 6, 8, 10, and 12 hours after infection. The samples were hybridized to HumanHT-12 v4 Expression BeadChip Kit (Illumina, San Diego, CA). Illumina arrays were log-transformed and quantile-normalized by using Lumi package [22]. The normalized expression levels were used to find activity of TFs and signaling pathways using QuSage [10, 20]. The target genes of TFs and the genes involved in the pathways were obtained from MSigDB [21]. Particularly, gene-sets for ligand induced signaling pathways were obtained from BIOCARTA, which had highest number of ligand induced pathways. The ligand induced signaling pathways and TFs which were found to be significant were used to assemble a network of causal relationships using MSigDB as performed in our previous studies [5, 21]. This is a first step towards construction of a Boolean model to study emergent properties of the signaling networks induced upon IV infection. Specifically, direct links were assembled to/from TFs to the ligand induced signaling pathway and between TFs. Direct link between two ligand induced signaling pathways was not allowed since its activation will require expression of a ligand. The network assembly and activity measurements were performed using the BioConductor software package in R [23].

### 2.2. Estimation of Logic Rules

To find the operational network defining underlying regulatory logic between the upstream regulators of each node in the network we identify a Boolean model that best fits the activities of TFs and signaling pathways. Since the kinetics and timescales of the individual processes represented as edges are not known, a random order asynchronous update was selected wherein the timescales of each regulatory process were randomly chosen in such a way that the node states were updated in a randomly selected order during each time-step [24]. The asynchronous algorithm was *X*_*i*_^*t*^ = *F*_*i*_(*X*_*a*_^*t*_*a*_^, *X*_*b*_^*t*_*b*_^, *X*_*c*_^*t*_*c*_^,…), where *F* is the Boolean transfer function, and *t*_*a*_, *t*_*b*_, and *t*_*c*_ represent the time points corresponding to the last change in the state of the input nodes *a*, *b*, and *c* and can be in the previous or current time-step. The time-step (time unit) of our model approximately corresponds to the difference in the time points at which experimental measurements were taken and is one hour. The randomized asynchronicity of the model does not alter the steady states of the dynamical system but causes stochasticity in the trajectory between the initial conditions and the equilibria (attractors) [24, 25]; thus, it can sample more diverse behaviors compared to traditional synchronous models. To determine the node consensus activity over time (i.e., shared by trajectories with different update orders) the simulations were run for 5–1000 times. The simulated activity profiles for each node were estimated by calculating the fraction of simulations in which the nodes were in* ON* state at each time-step. The simulated and observed activity profiles were compared using least-square method. The sum of least-squares error (SSE) values reached a plateau at 500 simulations, so for the further analysis activity profiles were calculated from 500 runs. The simulations were run by assigning initial state of all nodes based on the observations at 2 hours after infection. To find the optimum Boolean transfer functions two-stage procedure was used. First, asynchronous algorithm was run 10,000 times upon randomly choosing Boolean transfer functions from predefined percentage of* AND*,* OR*, and* NOT* logic rules. Particularly, 45%, 45%, and 10% were chosen for* AND*,* OR*, and* NOT*, respectively. The percentages were predetermined for robust results of the optimization. Second, the best operational network obtained from automated search algorithm for Boolean transfer functions was further simplified to ensure the minimum usability of* AND* and* NOT*. Particularly, in the absence of relevant perturbation data* AND* and* NOT* logic rules are not identifiable. As a first step towards simplification we replaced* AND* logic rules with* OR* logic rules iteratively. The changes that led to increase in the error were not kept. Similarly,* NOT* logic rules were also minimized. Additional simplification was performed to study the state-transition map (STM), which analyzes the evolution of a system over time starting from different initial conditions leading to identification of the steady states (attractors) of the system. Since the focus of the STM is to monitor long-term behavior chains of interactions can be collapsed into a single node, replacing the node with its upstream regulator when there is only one regulator. Furthermore, the minimum functional network was obtained by choosing a node with highest outdegree, when two regulators are connected by* OR* logic.

### 2.3. NFAT Inhibitor Treatment of IV-Infected DCs

Monocyte-derived DCs were obtained from a healthy human blood donor following a standard protocol [26]. Briefly, human peripheral blood mononuclear cells (PBMCs) were isolated from buffy coats in lymphocyte separation medium (Corning) by density gradient centrifugation at 1500 rpm. Then, CD14+ monocytes were purified by using a MACS CD14 isolation kit (Miltenyi Biotech). Monocytes were then differentiated into DCs by incubating the cells during 5 days, at 37°C, in growth media containing RPMI 1640 (Invitrogen/Gibco), 8% FBS (Hyclone), 2 mM glutamine, 100 U/mL penicillin, 100 g/mL streptomycin, 500 U/mL hGM-CSF (PeproTech), and 1000 U/mL hIL-4 (PeproTech). Before infection, DCs were pretreated for 1 h at 37°C with RPMI medium containing the NFAT inhibitors cyclosporine A (CsA, Sigma, 1 *μ*M) [27] or the VIVIT peptide (Tocris Biosciences, 100 *μ*M) [28]. Then, the cells were infected with the NC virus grown in embryonated chicken eggs as described previously [29], diluted in DMEM, and added directly to pelleted cells at a MOI of 1. After incubation of 40 minutes at RT, fresh RPMI medium containing CsA (1 *μ*M) or the VIVIT peptide (100 *μ*M) was added back, and the cells were incubated at 37°C during 2 h. Mock-infected cells underwent the same experimental procedure.

### 2.4. RNA Analysis by qRT-PCR

Total RNAs from mock- and IV-infected DCs were extracted at 2 hours postinfection (hpi) using the RNeasy mini kit (Qiagen). For quantitative reverse transcription PCR (qRT-PCR) of cellular and viral genes, cDNAs were synthesized using the High Capacity cDNA reverse transcription kit (Applied Biosystems) and random hexamers. The cDNAs were amplified by qPCR using Taqman gene expression assays specific for IL2 (Applied Biosystems, Hs-00174114_m1), GAPDH (Applied Biosystems, Hs-02786624_g1), and the IV gene M mRNA (Bei Resources NR-15594, 15595, and 15596). Data were acquired with an ABI PRISM 7300 sequence detection system (Applied Biosystems) and analyzed with ABI PRISM 7300 SDS version 1.0 software. Quantification was achieved using the 2^−ΔΔCt^ method [30].

## 3. Results and Discussions

### 3.1. TFs and Signaling Involved in IV Infection

**Figure 1:**
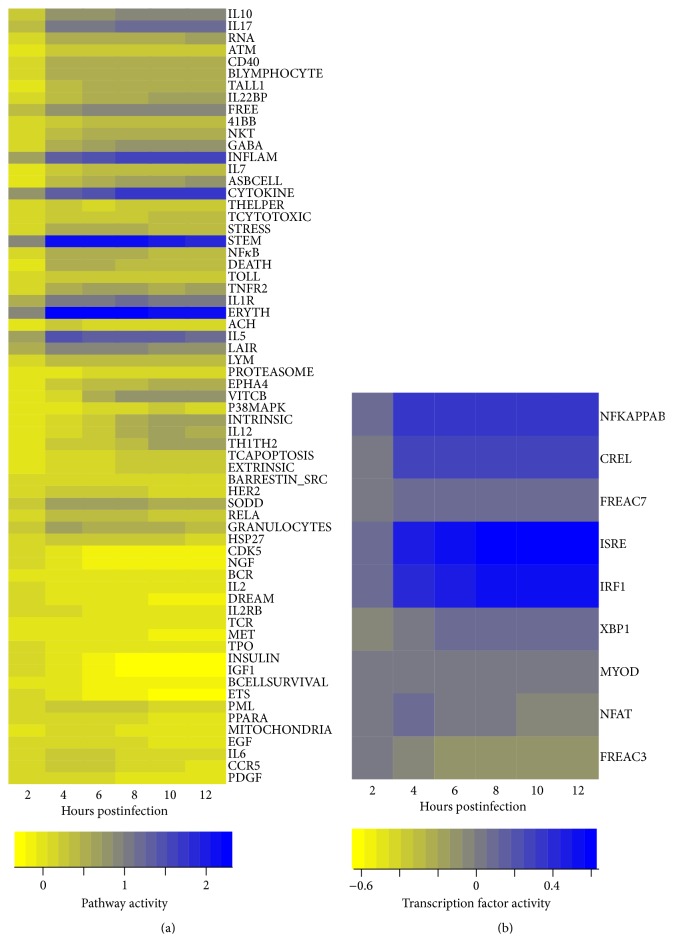
Signaling pathways' and transcription factors' (TFs) activities in response to IV infections: activities estimated by QuSAGE [20] using genome-wide expression profiles measured upon infections of DCs with NC virus, in comparison with mock-infected DCs. Activities are of (a) ligand induced signaling pathways from BIOCARTA database and (b) TFs using binding sites available in MSigDB [21] (rows), across time (*x*-axis). Coloring represents downregulated (yellow) to upregulated (blue) activity relative to the preinfection time point.

To detect ligand induced signaling pathways and TFs involved in antiviral response to IV infections of human monocyte-derived DCs we used a functional class scoring method. Specifically, QuSage was used to describe activities of ligand induced signaling pathways and TFs using gene-sets defined in MSigDB [21]. 81 signaling pathways and 9 TFs were significantly induced upon IV infections (Figure 1). Activity profiles of signaling pathways were clustered into two groups. The first group of pathways such as IL6, CCR5, PDGF, and EGF showed peak in their activity between 2 and 4 hours after infection. Activation of PDGF pathway leads to induction of several signal transduction pathways through the PI3K pathway or through reactive oxygen species-mediated activation of the STAT3 pathway [31]. Downstream effects of this include regulation of the cell cycle and cell migration [32] which are critical in generating adaptive immune response initiated by DCs. Similar to PDGF, the EGF pathway leads to cell proliferation, differentiation, and survival [33]. IL6 and CCR5 play a role in the inflammatory response to infection and have been associated with clinical outcomes during IV infection [34, 35]. Further, CCR5 plays a key role in CD8+ T cell responses [36]. The second group of pathways showed different activation profiles and included the majority of signaling pathways involved in the activation and differentiation of DCs, including NF*κ*B and p38MAPK [37, 38]. Additionally cytokine signaling pathways regulated by IL7 and IL1 were part of the second group [1, 9]. Relatively fewer TFs were detected. Specifically, NF*κ*B, cREL, IRF9, and IRF1 were increased over time of the infection, showing an increase in the activity similar to the second group of pathways. These TFs are involved in antiviral responses [38, 39]. NFAT and FREAC3 activity profiles were similar to the first group of pathways, showing a decline in activity at later time points.

### 3.2. Interaction Network of Cellular and Molecular Signaling

**Figure 2:**
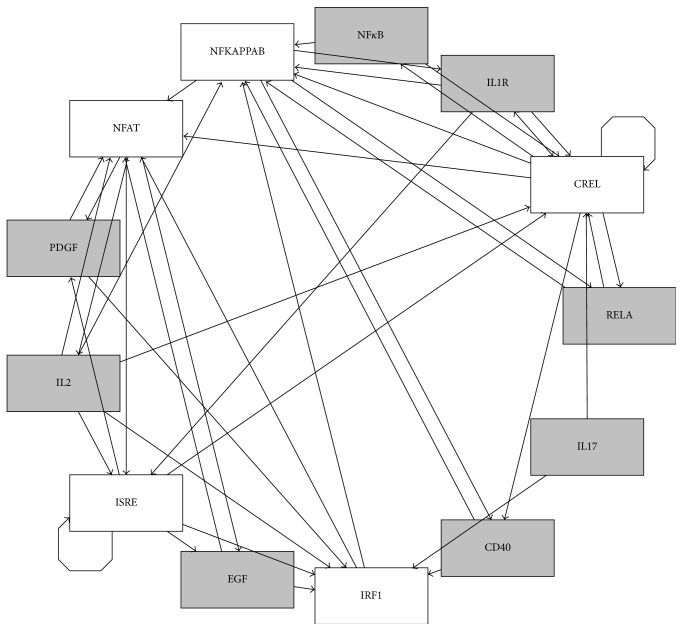
The network of interactions between TFs and ligand induced signaling pathways: rectangles represent network nodes and indicate the node name in an abbreviated manner. Terminating black arrows on an edge indicate causality but the sign of the effects (activation or inhibition) is unknown. Grey nodes represent signaling pathways and white nodes represent TFs.

To assemble the network of interactions between ligand induced signaling pathways and TF interactions we used MSigDB. Note that only ligand induced pathways were considered. For example, if NF*κ*B is in a gene-set for IL1 pathways, a directed edge was added from IL1 to NF*κ*B. Since ligand induced signaling pathways cannot induce another ligand induced signaling pathway without activation of a ligand any direct interaction between two ligand induced signaling pathways was not included. Several TFs and pathways were not included in the network due to absence of the source/upstream nodes. The resulting network consisted of 13 nodes; specifically five TFs regulated 8 signaling pathways (Figure 2) and 42 edges. It is likely that the nodes without known source nodes are directly induced by IV, which was considered while constructing the dynamic model (refer to next section). The graph in Figure 2 is represented using radial layout in which central position of NF*κ*B indicates that it has most interconnected paths in the network. The resulting network had 3 self-loops. IRF1 had highest indegree and outdegree indicating its critical role in the quick transfer of the signal upon viral infection. CREL had highest betweenness centrality implicating its role in connecting different signals. Moreover, CREL, NF*κ*B, and IRF1 had highest (>0.9) closeness centrality indicating cross talk between these TFs. In conclusion, the cellular and molecular network induced upon IV infection is a densely connected network, which has also been observed in other studies [5]. Perturbations of such densely connected networks could be ineffective due to a strong possibility of existence of alternative paths. This has been observed during the activation of antiviral responses, which constitutes induction of genes by NF*κ*B or IRF9 mediated pathways [5].

### 3.3. Boolean Dynamic Model Reveals Tight Regulation of IL2 by NFAT

**Figure 3:**
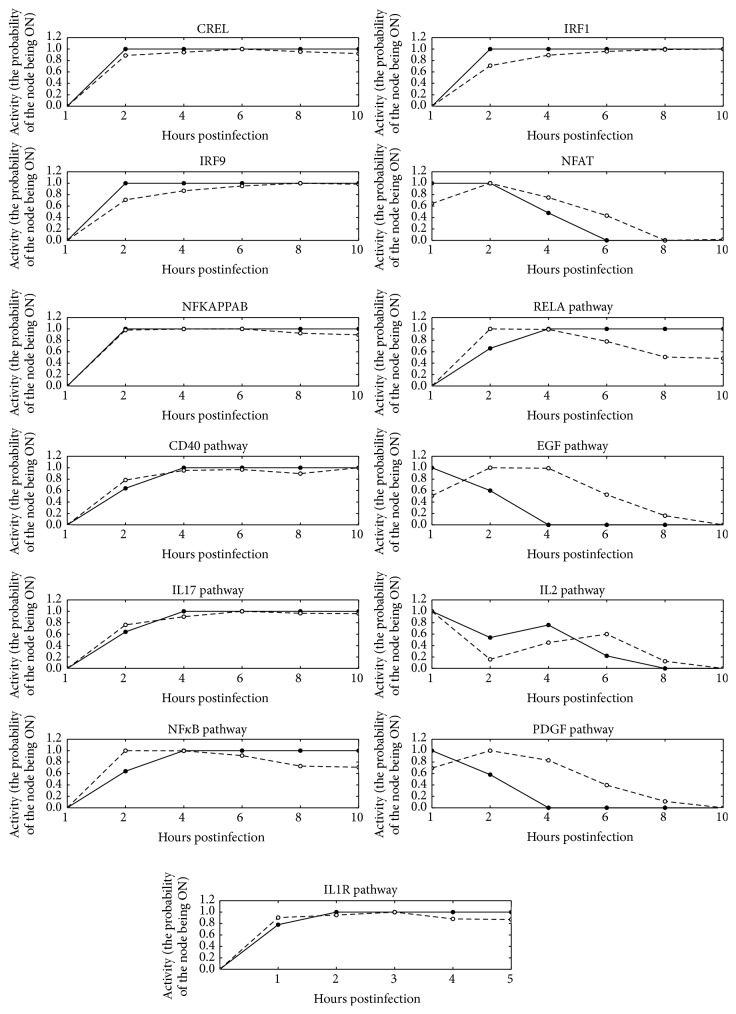
Results of the simulations of IV infection time course: activity profiles (*y*-axis) simulated (the probability of the node being in an ON state at a given time-step) by asynchronous model (solid lines and filled points) and estimated by QuSage using experimentally observed expression profiles (dashed lines and empty circles). Normalized expression profiles (*y*-axis) are normalized (0 to 1) and are plotted across hours after infection.

**Figure 4:**
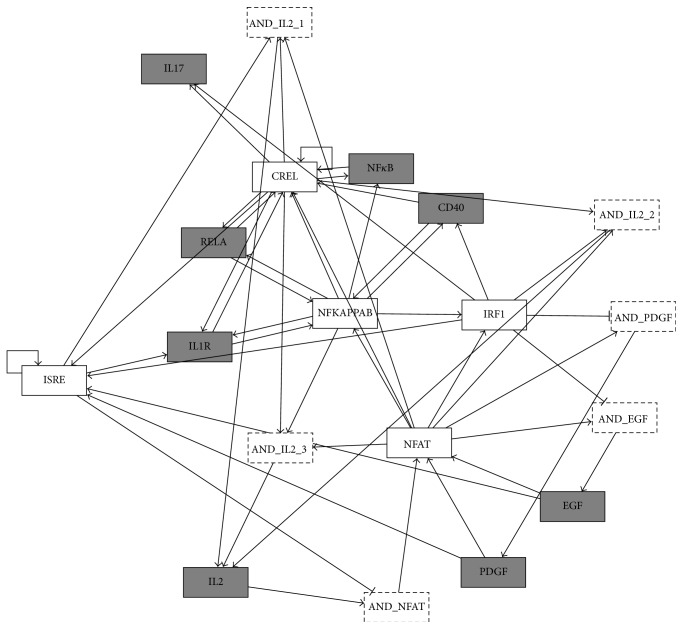
Enhanced regulatory network of cellular and molecular interactions: the network of interactions between ligand induced signaling pathways (grey rectangles with solid lines), TFs (white rectangles with solid lines), and their underlying logic controls (white rectangles with dashed lines). The connecting edges represent activating (line ending with black arrow) or inhibiting (line ending with black blunt segment) interaction.

**Figure 5:**
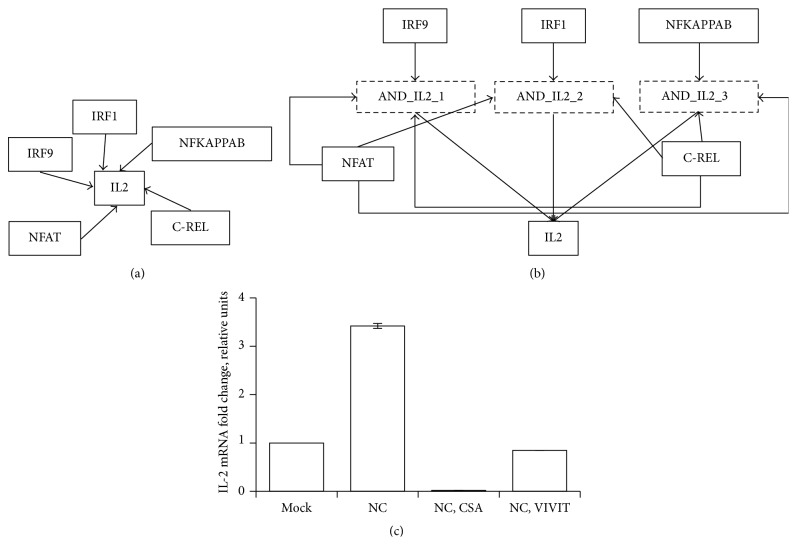
Regulation of IL2 by NFAT inferred by dynamic Boolean model: (a) network of interactions between IL2 and the TFs regulating its expression assembled from literature, (b) enhanced regulatory network inferred using dynamic Boolean model, and (c) the expression of IL2 mRNA evaluated by RT-PCR in DCs treated for one hour with medium containing the NFAT inhibitors CsA (CsA), VIVIT peptide (VIVIT), or vehicle (−), and then left mock-infected (mock) or infected with IV during 2 hours. The expression levels of IL2 in infected cells were normalized to the levels of nontreated, mock-infected cells.

The discrete dynamic Boolean model was developed to integrate temporal patterns (Figure 3 dashed lines) of the pathway and TF activities with the static interaction networks (Figure 2) assembled in the previous section. The Boolean models are parameter-free dynamic models which allow analysis of the sequence of events emerging into systems-level properties [17]. Development of dynamic model was facilitated by quantification of ligand induced signaling pathways and TF activities [20]. The Boolean model was developed using initial condition obtained from the experimentally observed initial state and regulators obtained from MSigDB. Specifically, PDGF, EGF, and IL2 signaling pathways and NFAT TF were set as ON in the initial state, which takes into account direct activation of molecular and cellular events induced by IV infection. Moreover, IRF1, IRF9, NF*κ*B, IL2, NFAT, and CREL were found to be the regulators of IL2 (Figure 4). The dynamic Boolean model was optimized by randomly sampling Boolean rules (see Section 2.2). The optimized model was simplified to find sparsest Boolean model that can describe the observed dynamics of ligand induced signaling pathways and TFs (Figure 4). Upon simplification, synergistic regulation was predicted between upstream regulators, for the nodes with asymptotic behavior such as CREL and IRF9. Interestingly, the Boolean model provided novel insight into the regulation of NFAT, IL2, PDGF, and EGF pathways that had complex temporal patterns. IL2 and NFAT are critical components of the adaptive immune response and are required for the activation, differentiation, and proliferation of T cells. The Boolean model revealed that IRF9 negatively regulated NFAT in the presence of IL2. Moreover, CREL and NFAT are required for the activation of IL2 when either one of NF*κ*B, IRF9, or IRF1 is present (Figure 5). Specifically, the IL2 rule was *IL*2^*∗*^ *= (IRF1 OR IRF9 OR NFκB OR IL2) AND NFAT AND CREL* (Figure 5(b)). Timely activation of IL2 by DCs must be critical for the activation and differentiation of T cells [40]. Novel insights were also provided in the regulation of PDGF and EGF signaling pathways, which were predicted to be inhibited by IRF1, and induced by NFAT. Note that the regulators are obtained from the literature [21]. Accordingly, promoter analysis involved the presence of NFAT-binding sites in EGF-mediated pathways [41]. The PDGF and EGF signaling pathways regulated the same set of TFs which in part is also explained by their shared functionalities during pathophysiological tissue remodeling and oncogenesis [42]. In conclusion, the Boolean model revealed logic controls underlying interaction network of cellular and molecular signaling involved in IV infection of DCs (Figure 4).

### 3.4. Validation of* AND* Logic in the Regulation of IL2 by NFAT

The Boolean model suggested that NFAT is required for the induction of IL2 during IV infection. To directly address the role of NFAT in IL2 signaling pathway, we treated IV-infected DCs that were treated with the two NFAT inhibitors, CsA, and the VIVIT peptide, which inhibit the phosphatase calcineurin, blocking NFAT activation [27, 28]. Two different inhibitors were used to minimize the likelihood of off-target effects. The levels of IL2 mRNA were measured. The IL2 mRNA increased at 2 hpi, in infected cells compared to mock-infected cells (Figure 5(c)), indicating that IV infection induces the expression of IL2 in DCs. Compared to nontreated cells, the levels of IL2 mRNA in CsA and VIVIT-treated cells were decreased by 171- and 4-fold, respectively (Figure 5(c)). As control, the expression of an NFAT-unresponsive gene (GAPDH) was not affected by the different treatments (data not shown). In addition, the treatment with the NFAT inhibitors did not affect the levels of IV replication, as the levels of viral gene M mRNA did not change significantly in nontreated and treated cells (Supplementary Figure  1 in Supplementary Material available online at http://dx.doi.org/10.1155/2016/7686081). These results indicate that the reduction in IL2 mRNA levels in NFAT inhibitor-treated cells is specific and is not simply due to a decreased IV replication. In conclusion, the experimental observations confirm the prediction of Boolean model, showing that NFAT is required for activation of IL2 pathway upon IV infection.

### 3.5. Functional Overlap between NF*κ*B and IRF Signaling

**Figure 6:**
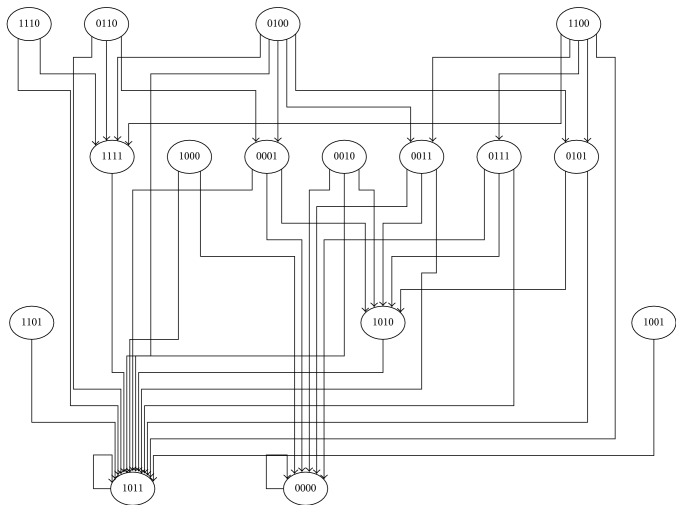
State-transition map of minimum functional network: attractor analysis of the minimal functional network reveals the transition from one state of the system (depicted by a node) to another state which is connected by an edge. The state of the system is defined by the states of each node in the minimal functional network which are depicted inside the nodes of the state-transition map. Particularly, 1/0 depicts the states of the nodes in the following order: c-REL, NF*κ*B, and PDGF pathways and IL1 pathway.

The inferred network can be simplified to find the parsimonious network structure. The parsimonious network structure depicts minimal functional network required to explain observed behavior. Such networks are often robust and optimize the costs and benefits of complexity. To find the minimum functional network induced in DCs upon NC virus infections, the network was simplified by selecting one of the upstream regulators based on their outdegree when they were connected by* OR* logic. The outdegree was used as a surrogate for measuring importance of the node in the network. The hypothesis was that highly critical nodes will translate the* OR* logic into a canalizing function. This simplification led to the selection of PDGF signaling pathway when both PDGF and EGF were upstream regulators connected by* OR* logic. Interestingly, several IV induced signaling events were regulated by NF*κ*B or IRF9. However, NF*κ*B regulated a large number of pathways leading to selection of NF*κ*B instead of IRF9 in the simplified network. Thus, the simplified network consisted of CREL, NF*κ*B, IL1, and PDGF. The state-transitions of these four nodes were analyzed to find attractors (steady states) attained by different initial conditions (Figure 6). Attractors of the Boolean networks typically describe the different states of the system following infections with IV. Two attractors of the system were found. The first attractor was defined by an* OFF* state for all four nodes, and the second attractor was defined by having NF*κ*B in an* OFF* state and all other nodes in an* ON* state. In conclusion, the attractor analysis suggests tight regulation of NF*κ*B.

## 4. Conclusions

Our results reveal the induction of a densely connected network of cellular and molecular signaling upon IV infection of DCs. This was accomplished by integrating known information on the interactions between pathways to reduce the dimension of genome-wide transcriptional profiles. To our knowledge this is a first study integrating gene-set enrichment methods with dynamic modeling. Development of a dynamic Boolean model reveals an operational network with underlying logic rules, and we experimentally validated the logic rule governing regulation of IL2 by NFAT. The model also reveals a critical role for NF*κ*B in delivering the antiviral response.

## Supplementary Material

Assessment of IV replication performed by measuring viral gene M mRNA.

## Abbreviations

NC: Influenza A/NC/20/1999
IV: Influenza virus
TFs: Transcription factors.

## References

[1] Law G. L., Korth M. J., Benecke A. G., Katze M. G. (2013). Systems virology: host-directed approaches to viral pathogenesis and drug targeting. *Nature Reviews Microbiology*.

[2] Fukuyama S., Kawaoka Y. (2011). The pathogenesis of influenza virus infections: the contributions of virus and host factors. *Current Opinion in Immunology*.

[3] Hartmann B. M., Thakar J., Albrecht R. A. (2015). Human dendritic cell response signatures distinguish 1918, pandemic, and seasonal H1N1 influenza viruses. *Journal of Virology*.

[4] Zaslavsky E., Hershberg U., Seto J. (2010). Antiviral response dictated by choreographed cascade of transcription factors. *Journal of Immunology*.

[5] Zaslavsky E., Nudelman G., Marquez S. (2013). Reconstruction of regulatory networks through temporal enrichment profiling and its application to H1N1 influenza viral infection. *BMC Bioinformatics*.

[6] Geiss G. K., Salvatore M., Tumpey T. M. (2002). Cellular transcriptional profiling in influenza A virus-infected lung epithelial cells: the role of the nonstructural NS1 protein in the evasion of the host innate defense and its potential contribution to pandemic influenza. *Proceedings of the National Academy of Sciences of the United States of America*.

[7] Go J. T., Belisle S. E., Tchitchek N. (2012). 2009 Pandemic H1N1 influenza virus elicits similar clinical course but differential host transcriptional response in mouse, macaque, and swine infection models. *BMC Genomics*.

[8] Huang Y., Zaas A. K., Rao A. (2011). Temporal dynamics of host molecular responses differentiate symptomatic and asymptomatic influenza a infection. *PLoS Genetics*.

[9] Korth M. J., Tchitchek N., Benecke A. G., Katze M. G. (2013). Systems approaches to influenza-virus host interactions and the pathogenesis of highly virulent and pandemic viruses. *Seminars in Immunology*.

[10] Thakar J., Hartmann B. M., Marjanovic N., Sealfon S. C., Kleinstein S. H. (2015). Comparative analysis of anti-viral transcriptomics reveals novel effects of influenza immune antagonism. *BMC Immunology*.

[11] Thakar J., Albert R. (2010). Boolean models of within-host immune interactions. *Current Opinion in Microbiology*.

[12] Christensen C., Thakar J., Albert R. (2007). Systems-level insights into cellular regulation: inferring, analysing, and modelling intracellular networks. *IET Systems Biology*.

[13] Kauffman S., Peterson C., Samuelssont B., Troein C. (2003). Random Boolean network models and the yeast transcriptional network. *Proceedings of the National Academy of Sciences of the United States of America*.

[14] Franke R., Müller M., Wundrack N. (2008). Host-pathogen systems biology: logical modelling of hepatocyte growth factor and *Helicobacter pylori* induced c-Met signal transduction. *BMC Systems Biology*.

[15] Thakar J., Pathak A. K., Murphy L., Albert R., Cattadori I. M. (2012). Network model of immune responses reveals key effectors to single and co-infection dynamics by a respiratory bacterium and a gastrointestinal helminth. *PLoS Computational Biology*.

[16] Thakar J., Pilione M., Kirimanjeswara G., Harvill E. T., Albert R. (2007). Modeling systems-level regulation of host immune responses. *PLoS Computational Biology*.

[17] Thakar J., Poss M., Albert R., Long G. H., Zhang R. (2010). Dynamic models of immune responses: what is the ideal level of detail?. *Theoretical Biology & Medical Modelling*.

[18] Thakar J., Saadatpour-Moghaddam A., Harvill E. T., Albert R. (2009). Constraint-based network model of pathogen-immune system interactions. *Journal of the Royal Society Interface*.

[19] Albert R., Thakar J. (2014). Boolean modeling: a logic-based dynamic approach for understanding signaling and regulatory networks and for making useful predictions. *Wiley Interdisciplinary Reviews: Systems Biology and Medicine*.

[20] Yaari G., Bolen C. R., Thakar J., Kleinstein S. H. (2013). Quantitative set analysis for gene expression: a method to quantify gene set differential expression including gene-gene correlations. *Nucleic Acids Research*.

[21] Liberzon A., Subramanian A., Pinchback R., Thorvaldsdóttir H., Tamayo P., Mesirov J. P. (2011). Molecular signatures database (MSigDB) 3.0. *Bioinformatics*.

[22] Du P., Kibbe W. A., Lin S. M. (2008). *lumi*: a pipeline for processing Illumina microarray. *Bioinformatics*.

[23] Gentleman R. C., Carey V. J., Bates D. M. (2004). Bioconductor: open software development for computational biology and bioinformatics. *Genome Biology*.

[24] Assmann S. M., Albert R. (2009). Discrete dynamic modeling with asynchronous update, or how to model complex systems in the absence of quantitative information. *Methods in Molecular Biology*.

[25] Saadatpour A., Albert I., Albert R. (2010). Attractor analysis of asynchronous Boolean models of signal transduction networks. *Journal of Theoretical Biology*.

[26] Fernandez-Sesma A., Marukian S., Ebersole B. J. (2006). Influenza virus evades innate and adaptive immunity via the NS1 protein. *Journal of Virology*.

[27] Henderson D. J., Naya I., Bundick R. V., Smith G. M., Schmidt J. A. (1991). Comparison of the effects of FK-506, cyclosporin A and rapamycin on IL-2 production. *Immunology*.

[28] Aramburu J., Yaffe M. B., López-Rodríguez C., Cantley L. C., Hogan P. G., Rao A. (1999). Affinity-driven peptide selection of an NFAT inhibitor more selective than cyclosporin A. *Science*.

[29] Steel J., Lowen A. C., Pena L. (2009). Live attenuated influenza viruses containing NS1 truncations as vaccine candidates against H5N1 highly pathogenic avian influenza. *Journal of Virology*.

[30] Livak K. J., Schmittgen T. D. (2001). Analysis of relative gene expression data using real-time quantitative PCR and the 2^−∆∆*C*_T_^ Method. *Methods*.

[31] Blaževic T., Schwaiberger A. V., Schreiner C. E. (2013). 12/15-Lipoxygenase contributes to platelet-derived growth factor-induced activation of signal transducer and activator of transcription 3. *The Journal of Biological Chemistry*.

[32] Yu J.-C., Li W., Wang L.-M., Uren A., Pierce J. H., Heidaran M. A. (1995). Differential requirement of a motif within the carboxyl-terminal domain of *α*-platelet-derived growth factor (*α*PDGF) receptor for PDGF focus forming activity chemotaxis, or growth. *The Journal of Biological Chemistry*.

[33] Herbst R. S. (2004). Review of epidermal growth factor receptor biology. *International Journal of Radiation Oncology, Biology, Physics*.

[34] Dawson T. C., Beck M. A., Kuziel W. A., Henderson F., Maeda N. (2000). Contrasting effects of CCR5 and CCR2 deficiency in the pulmonary inflammatory response to influenza A virus. *The American Journal of Pathology*.

[35] Shen Z., Chen Z., Li X. (2014). Host immunological response and factors associated with clinical outcome in patients with the novel influenza A H7N9 infection. *Clinical Microbiology and Infection*.

[36] Kohlmeier J. E., Miller S. C., Smith J. (2008). The chemokine receptor CCR5 plays a key role in the early memory CD8^+^ T cell response to respiratory virus infections. *Immunity*.

[37] Mikkelsen S. S., Jensen S. B., Chiliveru S. (2009). RIG-I-mediated activation of p38 MAPK is essential for viral induction of interferon and activation of dendritic cells: dependence on TRAF2 and TAK1. *The Journal of Biological Chemistry*.

[38] Wang X., Li M., Zheng H. (2000). Influenza A virus NS1 protein prevents activation of NF-*κ*B and induction of alpha/beta interferon. *Journal of Virology*.

[39] Nair S., Michaelsen-Preusse K., Finsterbusch K. (2014). Interferon regulatory factor-1 protects from fatal neurotropic infection with vesicular stomatitis virus by specific inhibition of viral replication in neurons. *PLoS Pathogens*.

[40] Macian F. (2005). NFAT proteins: key regulators of T-cell development and function. *Nature Reviews Immunology*.

[41] Wang J.-Y., Chen B.-K., Wang Y.-S. (2012). Involvement of store-operated calcium signaling in EGF-mediated COX-2 gene activation in cancer cells. *Cellular Signalling*.

[42] He H., Levitzki A., Zhu H.-J., Walker F., Burgess A., Maruta H. (2001). Platelet-derived growth factor requires epidermal growth factor receptor to activate p21-activated kinase family kinases. *The Journal of Biological Chemistry*.

