# Supplementary material for: Boolean Modeling of Cellular and Molecular Pathways Involved in Influenza Infection
Source: Comput Math Methods Med. 2016 Feb 14;2016:7686081. doi: 10.1155/2016/7686081 (PMC4769743; doi:10.1155/2016/7686081)
Supplement: Supplementary file 1 — Assessment of IV replication performed by measuring viral gene M mRNA. [file 7686081.f1.pptx]

## Slide 1
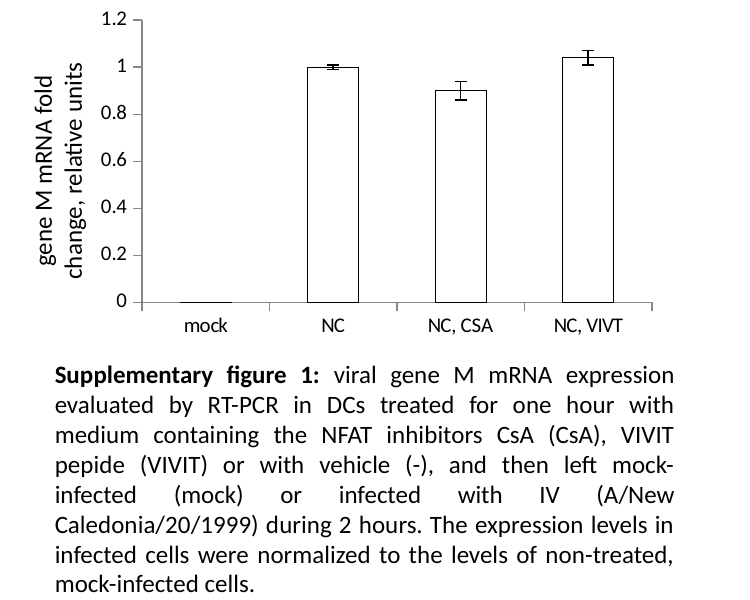

### Chart
| Category | |
|---|---|
| mock | 0.0 |
| NC | 1.0 |
| NC, CSA | 0.9 |
| NC, VIVT | 1.04 |gene M mRNA fold change, relative units
Supplementary figure 1: viral gene M mRNA expression evaluated by RT-PCR in DCs treated for one hour with medium containing the NFAT inhibitors CsA (CsA), VIVIT pepide (VIVIT) or with vehicle (-), and then left mock-infected (mock) or infected with IV (A/New Caledonia/20/1999) during 2 hours. The expression levels in infected cells were normalized to the levels of non-treated, mock-infected cells.
